# Uncertainty Quantification of Film Cooling Performance of an Industrial Gas Turbine Vane

**DOI:** 10.3390/e22010016

**Published:** 2019-12-22

**Authors:** Andrea Gamannossi, Alberto Amerini, Lorenzo Mazzei, Tommaso Bacci, Matteo Poggiali, Antonio Andreini

**Affiliations:** 1 Department of Engineering and Architecture, University of Parma, Via Università 12, 43121 Parma, Italy; 2 Department of Industrial Engineering, University of Florence, Via S. Marta 3, 50139 Firenze, Italy; alberto.amerini@htc.unifi.it (A.A.); lorenzo.mazzei@htc.unifi.it (L.M.); tommaso.bacci@htc.unifi.it (T.B.); matteo.poggiali@htc.unifi.it (M.P.)

**Keywords:** uncertainty quantification, CFD, gas turbine, blade, film cooling, polynomial chaos, Dakota

## Abstract

Computational Fluid Dynamics (CFD) results are often presented in a deterministic way despite the uncertainties related to boundary conditions, numerical modelling, and discretization error. Uncertainty quantification is the field studying how these phenomena affect the numerical result. With these methods, the results obtained are directly comparable with the experimental ones, for which the uncertainty related to the measurement is always shown. This work presents an uncertainty quantification approach applied to CFD: the test case consists of an industrial prismatic gas turbine vane with standard film cooling shaped holes system on the suction side only. The vane was subject of a previous experimental test campaign which had the objective to evaluate the film cooling effectiveness through pressure-sensitive paint technique. CFD analyses are conducted coherently with the experiments: the analogy between heat and mass transfer is adopted to draw out the adiabatic film effectiveness, solving an additional transport equation to track the concentration of CO_2_ used as a coolant fluid. Both steady and unsteady simulations are carried out: the first one using a RANS approach with k-ω SST turbulence model the latter using a hybrid LES-RANS approach. Regarding uncertainty quantification, three geometrical input parameters are chosen: the hole dimension, the streamwise inclination angle of the holes, and the inlet fillet radius of the holes. Polynomial-chaos approach in conjunction with the probabilistic collocation method is used for the analysis: a first-order polynomial approximation was adopted which required eight evaluations only. RANS approach is used for the uncertainty quantification analysis in order to reduce the computational cost. Results show the confidence interval for the analysis as well as the probabilistic output. Moreover, a sensitivity analysis through Sobol’s indices was carried out which prove how these input parameters contribute to the film cooling effectiveness, in particular, when dealing with the additive manufacturing process.

## 1. Introduction

Over the past years, the Turbine Inlet Temperature has progressively grown in order to improve the performance of the turbine in terms of thermodynamic efficiency and power output. Obviously, this leads to higher thermal loads and greater mechanical stress of all the components exposed to these high temperatures. For this reason, many efforts are dedicated to research and optimization of cooling systems trying to guarantee an extended lifetime for all working conditions and to minimize the coolant flow. This work is based on the test case studied and analyzed by Bacci et al. [1]. The adiabatic film effectiveness (η_ad_) was experimentally characterized in a previous campaign [2]. As demonstrated by previous works [3,4,5], the CFD analysis is a must-have tool for the design and the optimization phases of film cooled vanes and blades. However, CFD simulations are generally restricted to the situation where a set of constant input parameters are imposed, and a deterministic result is provided. In order to evaluate the effects of the input parameters variations, several stochastic approaches have been developed in recent years [6] and used in many fields of research [7]. The oldest and most common strategy is the Monte Carlo method; however, when dealing with computational expensive methodology, like high-fidelity CFD, other methodologies must be investigated. Recent research efforts have led to the development of several uncertainty quantification (UQ) methods [8], which can be classified as nonintrusive or intrusive, depending on whether the solver requires modification or not. In recent years, among these methodologies, polynomial chaos methods [9] (a nonintrusive technique) have had significant interest due to their high accuracy and computational efficiency compared to other methods. The stochastic expansion methods are based on the theory firstly developed by Wiener [10]. The first application was in mathematics on hypergeometric polynomials designed by Askey [11]. Regarding uncertainty quantification, the most interesting methods are the polynomial chaos expansion, developed by Kardaniakis-Xiu [12] based on the Askey’s scheme and the stochastic collocation. 

Furthermore, additive manufacturing techniques for gas turbine components have developed exponentially in recent years [13]. However, such techniques are affected by great sources of uncertainties both in the resulting dimensions of geometrical features and in the surface finishing of the part, largely affecting its aerothermal behavior and mechanical characteristics. Understanding the properties of the material deriving from additive manufacturing and the related UQ is, therefore, an important requirement. Nevertheless, only a few examples have been reported in the literature on UQ in AM [14,15]. Moreover, in the work presented by W. Shi [16], the effects of manufacturing, additive manufacturing, and electrical discharge machining (EDM) deviations on film cooling effectiveness provided by fan-shaped hole for a flat plate are considered.

## 2. Test Case 

**Figure 1:**
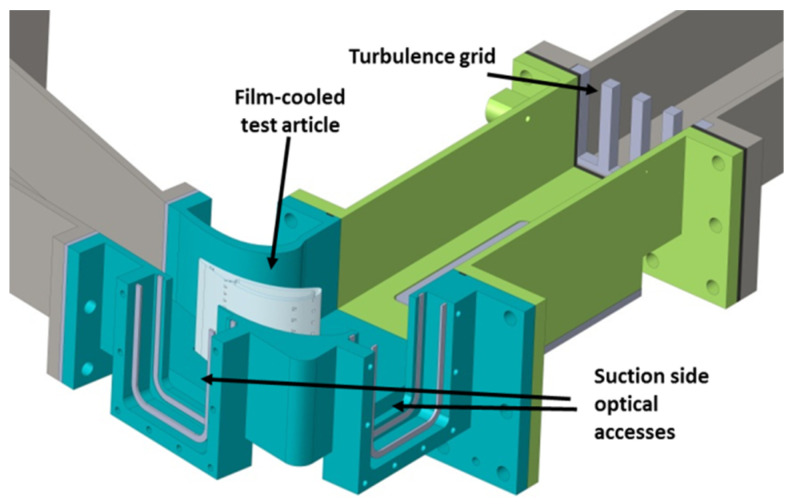
3D sectional view of the test rig.

The considered test article consists of a film-cooled prismatic vane airfoil, obtained through the extrusion of the midspan profile of a typical second stage vane. In a previous work [2], this airfoil was installed in a single vane, two-passages, linear cascade rig in order to experimentally characterize its behavior in terms of adiabatic film effectiveness. The cascade rig geometry (i.e., inlet duct, sidewalls, and outlet section) was designed through a CFD optimization process in order to prescribe representative conditions, in terms of Mach and Reynolds numbers as well as of flow acceleration and pressure gradients, within the two-passage configuration. A section of the test rig, with a focus on the test section, is reported in Figure 1. A more detailed description of the test apparatus can be found in [1,2].

**Figure 2:**
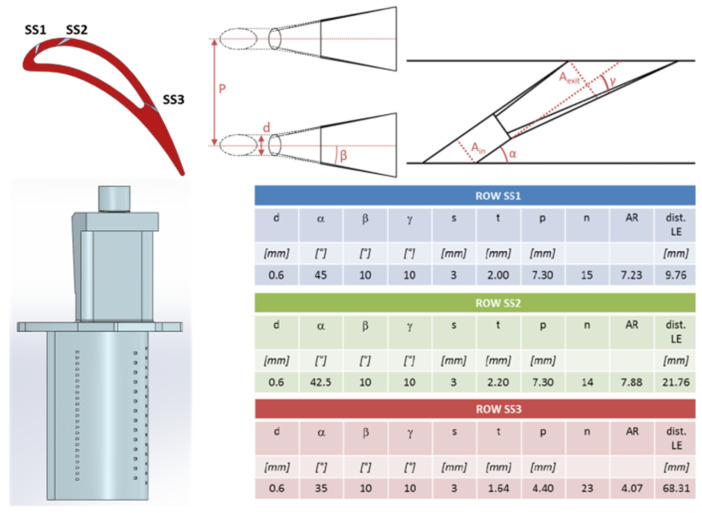
Test article geometry and cooling holes configuration [2].

The film cooling scheme adopted in the tested airfoil is characterized by three rows of axial laidback fan-shaped holes: two of them are located close to the leading edge (LE), while the third is far downstream and close to the trailing edge (TE). The cooling holes have a real engine diameter of 0.6 mm. All the geometric parameters of the cooling holes are reported in Figure 2, together with the airfoil profile and a CAD model of the whole test article. A cavity was realized inside the airfoil, in order to achieve a feeding-by-plenum configuration. The Inconel 718 test article was realized by 3D-printing (Direct Metal Laser Sintering with heat treatment), while the cooling holes were achieved by EDM. The adiabatic film effectiveness was evaluated by means of heat and mass transfer analogy, using the pressure-sensitive paint technique [17]. 

## 3. Numerical Methodology

In order to represent the experiment as faithfully as possible, the numerical analysis was also conducted based on the analogy between heat and mass transfer: two different fluids were used for the mainstream (air) and the coolant (CO_2_) and the effectiveness was evaluated simply considering the CO_2_ mass fraction on the vane surface. Given the objectives, it was decided to consider specific reference test conditions among those experimentally investigated: the one using CO_2_ as coolant with Blowing Ratio, BR = 1.

### 3.1. Geometry

**Figure 3:**
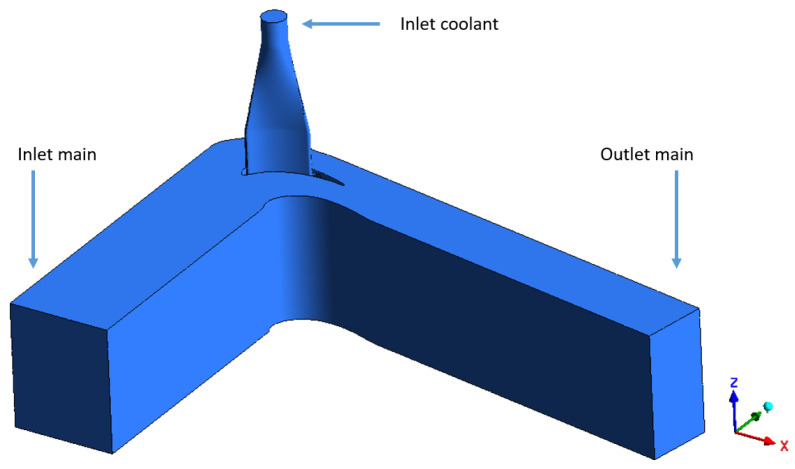
Numerical fluid domain.

Initially, the fluid domain considered was a full 3D representation of the test rig and it is shown in Figure 3. 

**Figure 4:**
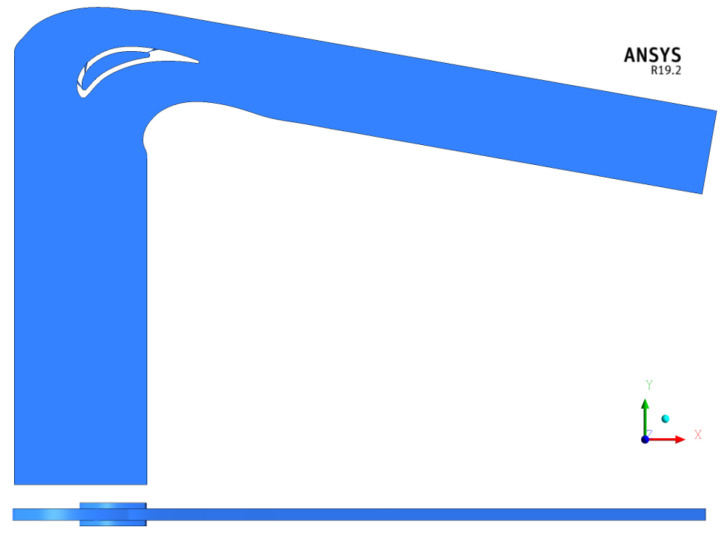
Reduced fluid domain (p/H = 7.3%).

Considering the huge computational effort required by the UQ analysis it was then decided to reduce the fluid domain in order to save computational resources. The minimum periodical portion (p/H = 7.3%) of the initial domain was considered with periodicity conditions on the upper and lower mainstream boundaries. Moreover, the distance of both inlet and outlet from the vane was reduced. Inlet and outlet boundary conditions were correctly imposed based on the results of the full 3D analysis. The reduced domain is presented in Figure 4. 

**Figure 5:**
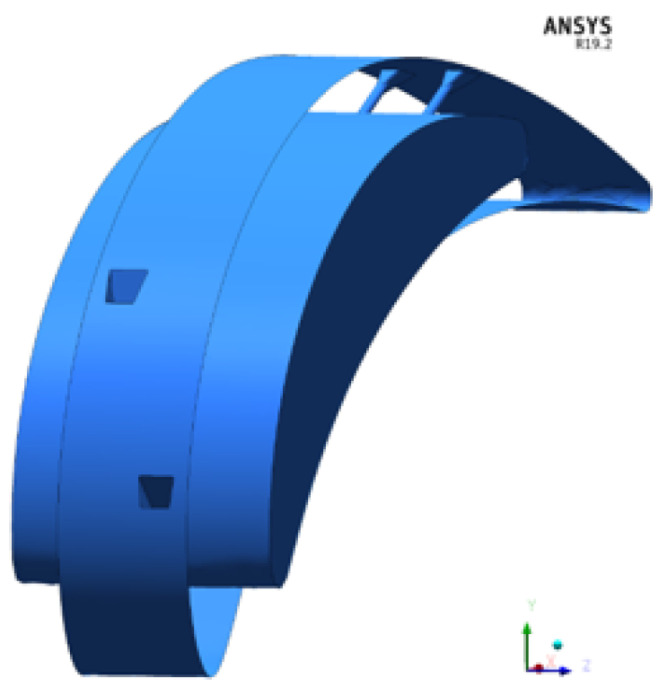
Vane section of the numerical domain adopted.

The domain was reduced to the minimum periodical conditions. However, the number of holes and their relative pitches did not allow for this. In order to obtain a full periodicity condition, the pitch of the third row of holes was reduced by 15%. It was verified that this adjustment, in accordance with the objective of the activities, had a minimum effect on the effectiveness. Regarding the coolant channel, a $\frac{p}{2}$ extension was added on the upper and lower boundary domain. These zones are not affected by periodicity and the lower boundary is a wall (i.e., the coolant mass flow is entirely exhausted by the film injection). Doing this, a more developed profile is guaranteed at the holes inlet. A closer view of the vane section used for the analysis is provided in Figure 5. 

### 3.2. Numerical Setup

ANSYS Fluent v19.2 was used for the analysis. RANS approach with k-ω SST turbulence model was adopted throughout this work. A second-order discretization was imposed for each quantity. Regarding the boundary conditions, a total pressure inlet and static pressure outlet were set as reported in Table 1, in accordance with the experimental measurements. Pressure conditions were imposed in order to allow for variation in mass flow and BR of the film cooling holes, in order to properly set the UQ analysis. Furthermore, with a reduced domain, this condition prevents errors in imposing the correct reduced mass flow at the inlet. It is important to notice that the inlet coolant channel acts as a plenum; therefore, the inlet pressure is constant throughout the height of the vane.

### 3.3. Mesh Sensitivity

Regarding the mesh, tetrahedral elements with 10 prismatic layers on the wall were adopted. A general sizing of $8\times 10^{-3}\;\mathrm{mm}$ was used, whereas for the suction side only, a refinement with $5\times 10^{-4}\;\mathrm{mm}$ was established. In fact, this will be the zone of major interest for the outputs. A mesh sensitivity considering three different grids was conducted: based on the mesh with the sizing provided before, a finer and a coarser mesh with a scale factor 1.5 was considered. Mesh characteristics are reported in Table 2. Approximately 100 CPU hours are required for the medium mesh in order for the solution to accurately converge.

**Figure 6:**
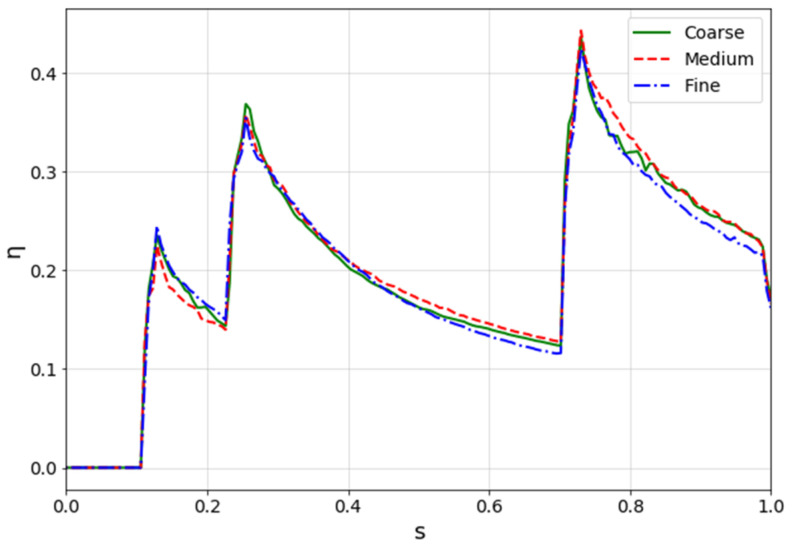
Mesh sensitivity results.

The comparison among the results is based on the adiabatic effectiveness on the suction side, averaged along the span-wise direction and presented in Figure 6. y+ is around 1 for the three meshes. 

**Figure 7:**
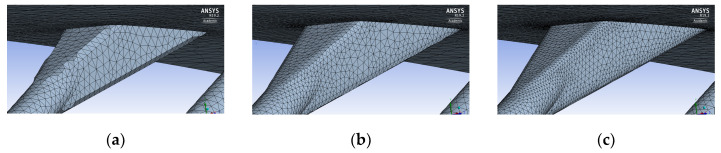
Mesh sensitivity hole detail; (**a**) coarse; (**b**) medium; (**c**) fine.

From the picture above, it is clear how results are approximately the same, even if the fine mesh performs slightly different in the last part of the suction side. However, for the scope of this activity, the medium mesh was chosen. In fact, even if the coarse mesh gives roughly the same results, it has a noticeable lack of spatial discretization (see Figure 7), especially along the edge of the holes, which can cause local numerical errors. 

### 3.4. Results

**Figure 8:**
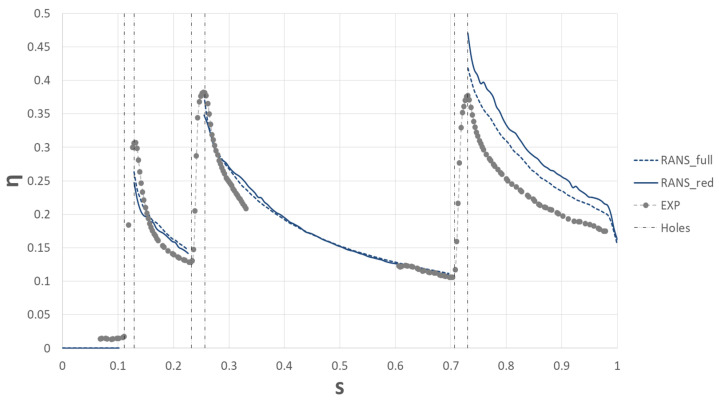
Result validation.

This section reports the results on which the upcoming UQ analyses, the main subject of this work, are based. Before moving to the UQ analyses, obtained results were compared and validated with the experimental data. Figure 8 shows the results from the experiments and the ones from the CFD analyses, both with the full domain and the reduced domain. Again, the results show the adiabatic effectiveness along the suction side of the vane, averaged on the spanwise direction between 35% and 65% of the span for the experiment, in order to avoid boundary effects. It is worth highlighting the absence of experimental data in the region 0.33 ≤ S ≤ 0.60, which is to be ascribed to the limitations to the optical access offered by the test rig for the IR visualization (see Figure 1).

**Figure 9:**
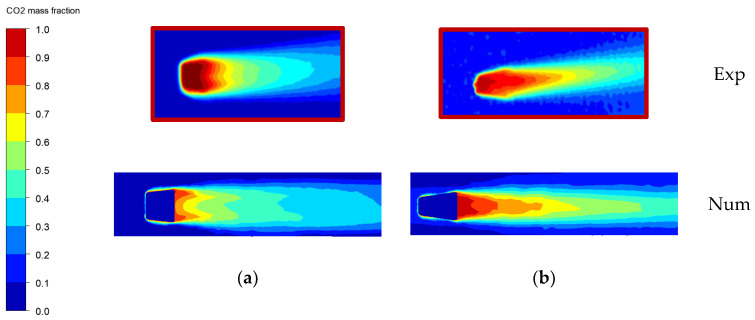
Hole details; (**a**) first hole; (**b**) third hole.

Generally, the results are in good agreement with those provided by the experiments. However, after the third row of holes, CFD overestimates the results by about 10%. It is believed that this discrepancy is due to the adopted steady RANS approach. After the third row of holes, the reduced domain overestimates the effectiveness with respect to the full domain by about 5%. As anticipated, this can be ascribed to the different pitch of the last row, which was reduced in order to obtain a periodicity condition. However, this discrepancy can be considered acceptable for the objective of this activity. From Figure 9a it appears that the first row of holes features a dual horn configuration, whereas the last row has a single horn configuration. This result is confirmed by the numerical simulations in Figure 9b. This is additional proof of the good quality of the results and it proves the solidity of the setup employed. 

### 3.5. Unsteady Approach

Even if this methodology could not be used for the uncertainty quantification analysis due to the high computational cost required, an unsteady approach was also adopted in order to show the capability of this methodology to model the film cooling effects. Despite the overall good capability of steady RANS to describe spanwise averaged adiabatic film effectiveness (as shown in Figure 8), an unsteady scale-resolving CFD method permits to better describe the mixing process of the injected film, offering a more depth insight in the involved physics.

A wall resolved LES approach would be unfeasible with the available resources; therefore, a hybrid RANS-LES approach was adopted. ANSYS Fluent was used also for this investigation, referring to a particular simulation setup: the Stress-Blended Eddy Simulation (SBES). Such an approach has been proposed by Frank and Menter [18] as further development of the Detached Eddy Simulation (DES) model. As other hybrid models, it is based on a dynamic blend between RANS and LES closures for the eddy viscosity [19] based on a shielding function f_s_:

(1) $$v_{t}^{SBES}=f_{s}\cdot v_{t}^{RANS}+\left(1-f_{s}\right)\cdot v_{t}^{LES}.$$

In order to prevent the use of LES subgrid model to all the zones, a shielding function was employed and a RANS approach was imposed in certain zones. Thus, a lower mesh resolution was needed, allowing to sensibly reduce the required computational effort. As far as the RANS approach is concerned, a k−ω SST turbulence model was adopted, while for the unsteady approach a dynamic Smagorinsky subgrid-scale model was employed [20,21,22].

Regarding the mesh, an additional sensitivity analysis was performed and the final selected mesh features approximately 32 million elements. The differences with the previous one are 40 prismatic layers near the wall instead of 10 and a defeature size of $1\times 10^{-4}\;\mathrm{mm}$ instead of $5\times 10^{-4}\;\mathrm{mm}$. A total of 13,000 CPU hours are approximately required in order for the SBES simulation to converge. This is additional proof that this approach is not feasible for the uncertainty quantification analysis.

**Figure 10:**
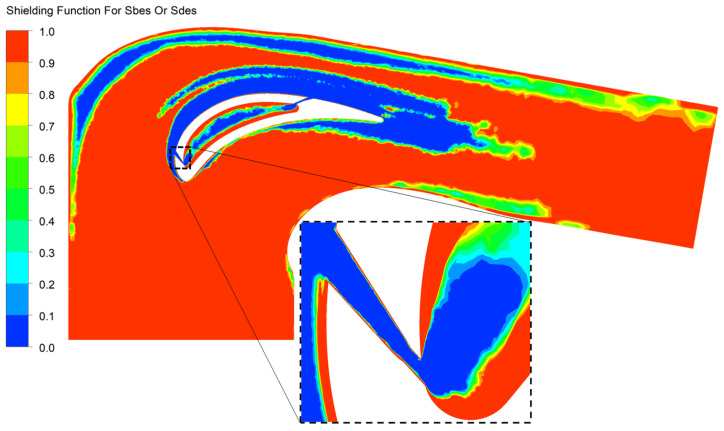
Shielding function for 32M elements mesh (SBES) simulation.

Concerning the results, the first aspect to point out is the shielding function, defined in Equation (1), which could assume values between 0 (where it solves for LES equations) and 1 (where it solves for RANS equations). Figure 10 provides the shielding function of this particular test case. Even if the largest part of the domain is treated with an eddy viscosity turbulence model, including the inlet zone, the region surrounding the vane, and especially the suction side, are solved with a LES approach in the freestream. The fact that the inlet is solved with a RANS approach and the turbulence is therefore modelled (rather than solved by LES) should not have a significant impact on the results. Preliminary sensitivity analyses carried out in a purely RANS approach, not reported for the sake of brevity, proved how the inlet turbulence level has almost no effect on the film cooling effectiveness on the vane surface. The vicinity of the first row to the LE causes a wide mixing of the flow immediately after the hole.

**Figure 11:**
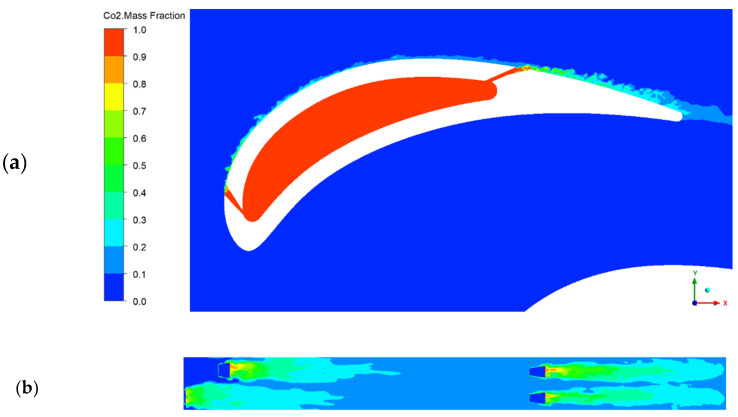
Instantaneous CO_2_ mass fraction. (**a**) Cross-sectional view of the first and third hole; (**b**) suction side.

Figure 11 provides some snapshots of the instantaneous distribution of CO_2_ mass fraction on a cross-sectional plane that cut row 1 and 3 (11a), and for the suction side of the vane (11b). Evident unsteady behavior can be observed near the exit of the hole, increasing towards the TE. 

**Figure 12:**
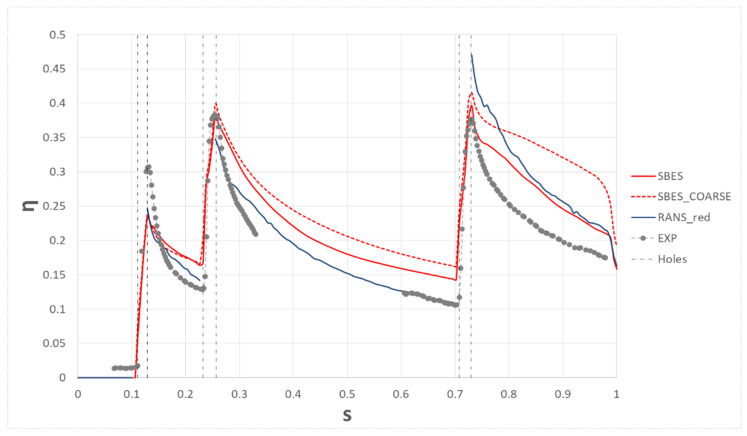
Average film cooling effectiveness profiles on the suction side.

Regarding the average film cooling effectiveness on the suction side, results are provided in Figure 12. The parameter was evaluated for four different cases: the unsteady approach with the 32M elements mesh (SBES), the unsteady approach with the same mesh as the RANS approach (SBES_COARSE), the steady approach RANS, and the experiment. The first thing to notice is the importance of having a proper mesh size for the unsteady approach: in fact, the finer the mesh, the better the approach can solve the turbulence and capture the smaller scales. The second thing to look at is that the SBES approach did not perform as expected: in fact, if compared to the RANS approach the SBES model shows a general overestimation. However, the mesh refinement seems greatly beneficial, probably due to a reduction of the numerical dissipation that reduces the turbulent mixing between coolant and mixing. Despite the already high computational effort, it is clear that the mesh sizing is not small enough to provide a good accuracy, therefore, given the objectives of the present work, it is clear that the RANS approach is adequate to carry out the following UQ analysis.

## 4. Uncertainty Quantification

The objective of the present work was to conduct a UQ analysis based on three input geometric uncertainties and to investigate the effect on the film effectiveness, the BR, and the C_d_. The uncertainties considered concern the geometry of the holes: inlet fillet radius, a scale factor, and streamwise inclination angle were considered and the analysis was carried out with the open-source code Dakota [23].

### 4.1. Methodology

As far as the UQ methodology is concerned, a polynomial chaos approach with probabilistic collocation method was used. It is beyond the scope of the current paper to present in detail all the mathematical information of the methodology. For an in-depth analysis, the authors suggest looking at the works presented in the introduction, as well as in Dakota’s manual [23]. Only a basic overview is presented in the following lines.

A stochastic function, R(x,ξ), can be expressed by a series expansion:

(2) $$R\left(x,\xi \right)=\overset{\infty }{\underset{i=1}{\sum }}\alpha_{i}\left(x\right)\psi_{i}\left(\xi \right),$$

where $\alpha_{i}\left(x\right)$ are deterministic coefficients of the polynomials and $\psi_{i}\left(\xi \right)$ is the orthogonal basis, which are functions of the probabilistic distributions of the input variable. Equation (2), in practice, is always truncated at a finite expansion order [23]:

(3) $$R\left(x,\xi \right)≅\overset{P}{\underset{i=1}{\sum }}\alpha_{i}\left(x\right)\psi_{i}\left(\xi \right).$$

When the order “$p_{i}$” of each polynomial basis $\psi_{i}$ is fixed, the approach is called “tensor-product expansion”, the one used in this work. The number of evaluations $N_{t}$ depends on the order “p” of polynomial and the number of variables “n”. The total number of evaluations is given as follows

(4) $$N_{t}=1+P=\overset{n}{\underset{i=1}{\prod }}\left(p_{i}+1\right).$$

The input points are fixed and defined by the Gauss grid. Tensor-product approach is excellent for a small number of input variables. For a large number of variables, other approaches are preferable. A first order approximation in each variable was adopted (global polynomial order = 3), resulting, from Equation (4), in a total of eight required evaluations. This methodology is able to reproduce accurately the results coming out from the input domain space that a usual Monte Carlo analysis provides with thousands of simulations. 

### 4.2. Input Uncertainties

Three input uncertainties were considered for the analysis: streamwise angle, inlet fillet radius, and the sizing. 

The streamwise angle has never been considered in the open literature related to UQ studies about film cooling. According to Bunker [24], a Gaussian distribution N(ref, 2.5°) truncated at ±5° (±2σ) was chosen for the uncertainty. This uncertainty was set the same for all the holes with respect to their nominal angles. The origin for the rotation was placed on the final part of the hole, on the suction side of the vane. This choice was made in consideration of the EDM manufacturing process. In fact, this process can be extremely precise; however, if the vane has a shape different from the nominal one, the real hole profile can be completely different than expected. In order to partially deal with this problem, usually, during an EDM process, a laser scan is performed in order to ensure the correct initial location of the hole.

Fillet radius has already been investigated by Montomoli et al. [25]. Following this study, a Gaussian distribution for the r/D N(5%, 2%) truncated at 1% and 9% (±2σ) was adopted. The case with no fillet radius was not included for the physical consistency of the results: in fact, the simulations required in order to build the polynomial chaos method depend on the Gauss grid, which does not include the boundary extreme values. Therefore, a nonphysical result can occur when trying to represent a non-fillet condition using fillet-holes simulations. 

Regarding the scale factor, again, for this variable a Gaussian distribution N(1, 0.05) truncated at 0.9 and 1.1 (±2σ) was chosen.

### 4.3. Results

The eight simulations required by the tool to build the polynomial chaos approach and based on the Gauss grid are presented in Table 3.

Once the eight different results were obtained, the polynomial chaos methodology could be implemented using Dakota. Uncertainty quantification results involve the adiabatic effectiveness, the blowing ratio (BR), and the discharge coefficient (C_d_). The adiabatic film effectiveness η_ad_ was evaluated as the CO_2_ concentration on the vane surface. As for the previous chapter, results were averaged along the spanwise direction. 

**Figure 13:**
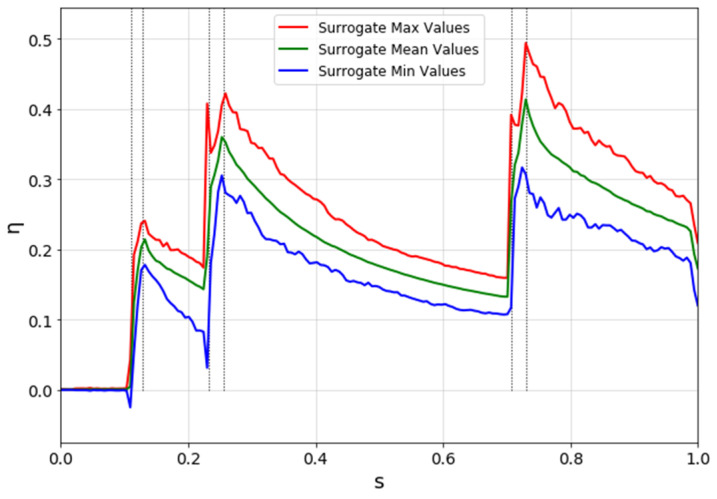
Uncertainty quantification (UQ) results for η_ad._

Figure 13 shows the output in terms of mean, maximum, and minimum values as a function of the nondimensional abscissa on the suction side. Results, for each nondimensional abscissa, are independent of each other: in fact, the nondimensional abscissa was discretised in 1000 parts, and for each part, a surrogate (polynomial chaos) was created.

**Figure 14:**
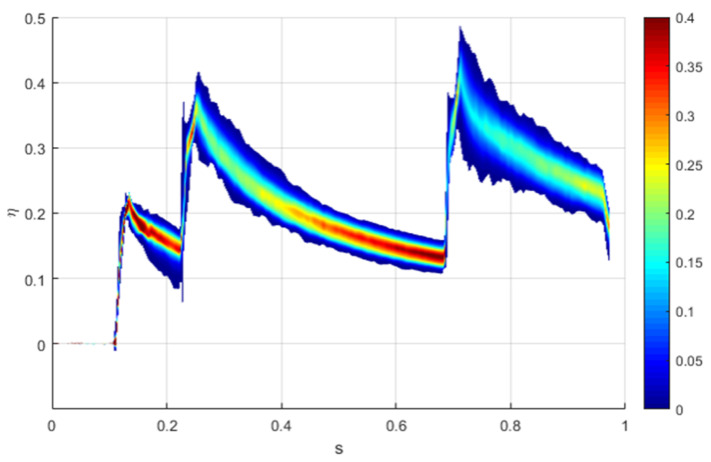
Probability Density Function for η_ad._

Figure 14 provides the Probability Density Function (PDF) for the η_ad_. This graph is able to show not only the range but also the probability associated with each zone within this range. It is clear how the zones immediately after the second and the third rows are affected by a greater uncertainty: in fact, not only the range maximum-minimum is almost the highest, but also the color is more uniform than other zones, meaning that the probability is approximately the same for each η_ad_ of that particular nondimensional abscissa.

After the third row, uncertainty can reach up to 20%. On the contrary, the zone after the first hole and from s ≈0.5 to s ≈0.68 has a high-probability zone at the center of the range: this means that, even if the range can be the same, the uncertainty is lower. 

**Figure 15:**
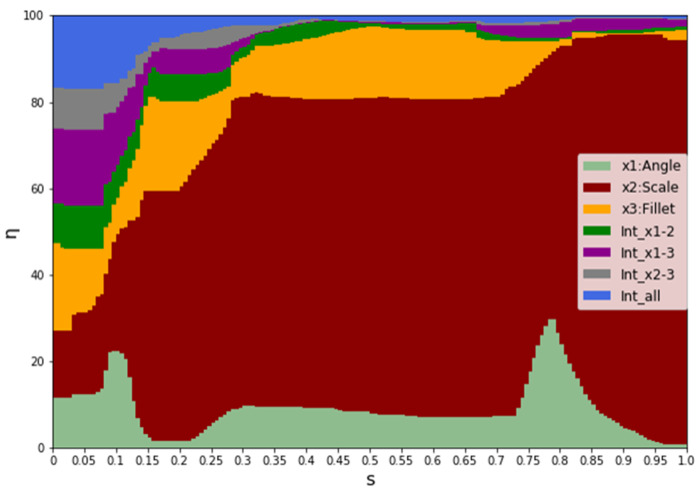
Sensitivity analysis for η_ad_.

In order to have a complete outline of the results, a sensitivity analysis through Sobol’s indices was carried out. Results are depicted in Figure 15. Main Sobol’s indices, or first order indices, were used for the graph: the first three indices are for the variables, the other four represent all the possible interactions. The graph is a cumulative histogram: the influence of each parameter is represented by its relative colored stripe. Results are consistent after s = 0.1, where the film cooling starts its effect. The scale factor dominates, especially in the last part of the suction side. The angle has a moderate influence on the first and the third holes; for the second hole the influence is lower: this is probably due to the location of the hole and the influence of this angle on the inlet area of the perforation. Interactions do not affect the results by more than 10% in the zone of interest. 

**Figure 16:**
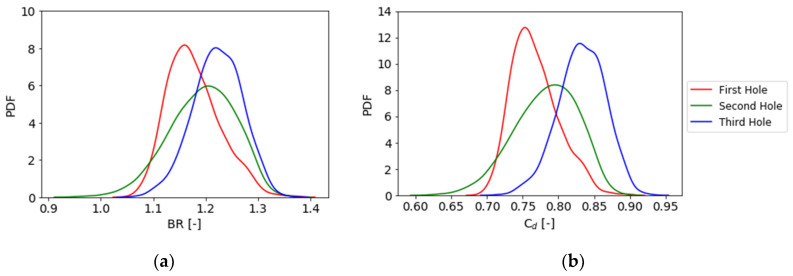
Blowing ratio (**a**) and C_d_ (**b**) Probability Density Function (PDF) for the different holes.

To calculate the BR and C_d_, it was required to calculate the pressure and the isentropic Mach number on the vane surface. Therefore, a simulation without film cooling was carried out. BR is calculated with the following equation:

(5) $$BR=\frac{{\left(\rho V\right)}_{coolant}}{{\left(\rho V\right)}_{\infty }},$$

where *ρ* is the density of the fluid, *V* the velocity and ∞ stands for the isentropic condition on the vane surface, obtained from a CFD simulation without film cooling. C_d_ is calculated with the following equation:

(6) $$C_{d}=\frac{\dot{m}}{P_{0,c}{\left(\frac{P_{\infty }}{P_{0,c}}\right)}^{\frac{\gamma +1}{2\gamma }}\sqrt{\frac{2\gamma }{\left(\gamma -1\right)RT_{0,c}\;}\left({\left(\frac{P_{0,c}}{P_{\infty }}\right)}^{\frac{\gamma -1}{\gamma }}-1\right)}\frac{\pi }{4}D^{2}\;\;},$$

where the subscripts “*c*” and “∞” stand for coolant and mainstream conditions, *P*_0_ and *P* are total pressure of the coolant inlet and static pressure at the outlet, and D is the hole minimum diameter. Results of the UQ analysis for the BR are presented in Figure 16a. It is clear how the BR has a modal value which is not the same among the three rows, but close to 1. The second hole is the one that presents a bigger uncertainty: in fact, the Probability Density Function has a lower and wider trend, which means that it is more spread out with space. Results and considerations for the C_d_ are approximately the same and presented in Figure 16b.

**Figure 17:**
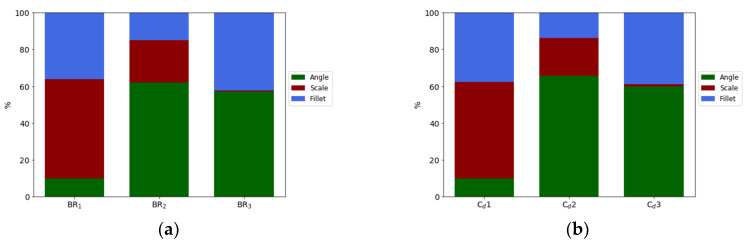
Sobol’s indices for BR (**a**) and C_d_ (**b**).

A sensitivity analysis for these two parameters was performed and results are presented in Figure 17. It is clear how the most influencing parameters vary depending on the hole location and the parameter taken into consideration. Considering the physical dependencies between BR and C_d_, it is not a surprise that the results, as shown by the PDFs, are similar between these two parameters. The scale factor has a major effect on the first hole where the pressure drop is lower, whereas it gradually decays going towards the trailing edge. For the second row of holes, the parameters that have the greatest effect on the result is the angle, the opposite as shown for the η_ad_. For the third row, the scale factor has almost no effect because of the higher pressure drop on the holes; here the streamwise angle and the fillet radius play, respectively, for 60% and 40% of the final outcomes.

## 5. Conclusions

The objective of this activity was to carry out an uncertainty quantification analysis on an additive manufactured gas turbine vane. Initially, results were validated with the experimental ones provided by Bacci et al. [2]. This part proved how a RANS simulation with k-w SST turbulence model with a full domain accurately represents the experimental outcomes, except for the last row with a 10% overestimation in terms of η_ad_. A reduced domain was considered, which causes a discrepancy of around 5% in η_ad_ due to the reduced pitch of the last row of holes. Moreover, an unsteady analysis using a hybrid LES-RANS approach was also investigated. The SBES option allowed to switch from a RANS approach of low-interest zones to a LES approach of the relevant zone. A total of 13,000 CPU hours are required by the unsteady approach, whereas only 100 CPU hours are required by the RANS approach. The results, however, due to the high isotropy of the turbulence, are not as good as expected and the RANS simulation was adopted for the statistical analysis. An uncertainty quantification analysis using a polynomial chaos approach was performed on this test case. A probabilistic collocation method based on the Gauss grid was adopted. Three variables (streamwise angle, scaling factor, and fillet radius) were investigated and the analysis required eight simulations. This analysis was able to accurately represent what a classic Monte Carlo analysis does with thousands of simulations. The complex phenomenology and the different shape among the three holes do not allow to draw a general conclusion for this part. However, it is clear how the scaling factor accounts for 60% of the effectiveness, whereas the angle accounts for 20% only in the vicinity of the first and third hole. The closer to the trailing edge, the higher the influence of the scaling factor on the η_ad._ As expected, BR and C_d_ are in accordance. Here the scaling factor has an opposite trend with respect to the η_ad_: it has a great influence for the first hole (around 55%) and almost no influence on the last hole. For the second hole, the parameter that has the highest influence is the angle (60%) and this is probably due to the location and the shape of the vane.

## Figures and Tables

**Table 1:** Boundary conditions.

Inlet Mainflow	P_0_ = 135,770 Pa	T_0_ = 286.15 K
Outlet Mainflow	P_0_ = 123,279 Pa	
Inlet Coolant	P_0_ = 144,760 Pa	T_0_ = 286.15 K

**Table 2:** Mesh characteristics.

	Coarse	Medium	Fine
N° Elem	2.06 M	3.18 M	4.33 M
N° Nodes	635 k	972 k	1.33 M

**Table 3:** Evaluations.

	Angle	Scale	Fillet Radius
**1**	−2.2°	0.956	2.7%
**2**	2.2°	0.956	2.7%
**3**	−2.2°	1.044	2.7%
**4**	2.2°	1.044	2.7%
**5**	−2.2°	0.956	7.3%
**6**	2.2°	0.956	7.3%
**7**	−2.2°	1.044	7.3%
**8**	2.2°	1.044	7.3%
